# The prevalence and clinical correlates of substance use disorders in patients with psychotic disorders from an Upper-Middle-Income Country

**DOI:** 10.4102/sajpsychiatry.v26i0.1473

**Published:** 2020-07-28

**Authors:** Henk S. Temmingh, Sumaya Mall, Fleur M. Howells, Goodman Sibeko, Dan J. Stein

**Affiliations:** 1 Department of Psychiatry and Mental Health, University of Cape Town, Cape Town, South Africa; 2 Division of Epidemiology and Biostatistics, School of Public Health, University of the Witwatersrand, Johannesburg, South Africa; 3 Neuroscience Institute of South Africa, Cape Town, South Africa; 4 MRC Unit on Risk & Resilience in Mental Disorders, University of Cape Town, Cape Town, South Africa

**Keywords:** substance use, schizophrenia, psychosis, clinical correlates, low-and-middle-income countries

## Abstract

**Background:**

Substance use disorders (SUDs) occur frequently in patients with psychotic disorders and have been associated with various demographic and clinical correlates. There is an absence of research on the prevalence and clinical correlates of SUDs in psychotic disorders in low-and-middle-income countries (LMICs).

**Aim:**

We aimed to determine the prevalence and correlates of SUDs in psychotic disorders.

**Setting:**

Patients attending a large secondary-level psychiatric hospital in Cape Town South Africa.

**Methods:**

We used the Structured Clinical Interview for DSM-IV (SCID-I) to determine psychiatric and substance use diagnoses, depressive, anxiety, obsessive-compulsive and post-traumatic symptoms. We used logistic regression models to determine significant predictors of SUDs.

**Results:**

In total sample (*N* = 248), 55.6% of participants had any SUD, 34.3% had cannabis use disorders, 30.6% alcohol use disorders, 27.4% methamphetamine use disorders, 10.4% methaqualone use disorders and 4.8% had other SUDs. There were significant associations with male sex for most SUDs, with younger age and Coloured ethnicity for methamphetamine use disorders, and with lower educational attainment for cannabis use disorders. Anxiety symptoms and suicide attempts were significantly associated with alcohol use disorders; a diagnosis of a substance induced psychosis with cannabis and methamphetamine use disorders. Across most SUDs legal problems and criminal involvement were significantly increased.

**Conclusion:**

This study found a high prevalence and wide distribution of SUDs in patients with psychotic disorders, consistent with previous work from high income countries. Given clinical correlates, in individuals with psychotic disorders and SUDs it is important to assess anxiety symptoms, suicidality and criminal involvement.

## Introduction

Depending on the sample characteristics and setting, the prevalence of substance use disorders (SUDs) in patients with serious mental disorders varies from as low as 10% to as high as 74%.^1,2,3^ Whereas the variation in prevalence is affected by a variety of factors, meta-analyses of prevalence studies report cannabis use disorders to occur in 27.1%, alcohol use disorders in 20.6% and amphetamine use disorders in 10.4% of patients with major affective and non-affective psychoses.^4,5,6^ SUDs have a negative impact on the clinical course and outcome of patients with SMI, and higher rates of re-hospitalisation and poor clinical outcomes are reported in patients with SUDs.^2,7,8^

Variation in the prevalence of difference substance use disorders is influenced by factors such as geographical region, setting, phase of illness (first vs. chronic), diagnostic method and other demographic and clinical characteristics. Some of these clinical characteristics include variations in clinical diagnosis and ethnic grouping, and in some studies stimulant use disorders were more prevalent in patients with affective psychosis,^6^ and some but not all studies have shown higher prevalence in patients from some ethnic groups.^9,10,11^ In turn, whereas meta-analyses and studies with predominantly schizophrenia spectrum patients report the most prevalent substance used to be cannabis or alcohol, followed by stimulants such as cocaine and amphetamines,^1,4,5,6,12,13,14,15,16,17,18,19,20^ some studies of predominantly bipolar patients report alcohol use disorders to be most prevalent.^21^

In patients with psychotic disorders and SUDs, substance rehabilitation treatment is typically reported as to be low.^22,23^ In the South African setting, where community mental health teams are severely overburdened and under-resourced, most patients with co-occurring SMI and SUDs are treated in the public mental health sector, mostly as inpatients where clinical presentations are characterised by severe relapses of mental disorder, and treatment tends to be sequential with mental health treatments occurring first, and only a small proportion of patients then getting referred on to traditional drug and alcohol rehabilitation centres in the community. In one SA sample less than 5% of SMI/SUD patients had attended some form of substance rehabilitation program.^3^ Pressured mental health services may be forced to discharge such patients as soon as the mental illness has been stabilised, often leaving patients in a pre-contemplative phase regarding change in terms of substance use.

Whereas most studies report that male sex, younger age, ethnic minority status, low educational attainment, unemployment, single marital status are significantly associated with SUDs,^1,3,10,17,18,24,25,26,27,28,29,30,31,32,33^ a few studies have shown a lack of these associations.^15,19^ Patients with co-occurring SUDs are also more likely to have contact with law enforcement with subsequent arrests, in particular for minor and drug related crimes.^1,23,30,34,35,36,37^ The association between dual diagnosis and mood or anxiety symptoms vary, with some studies reporting lower mood, anxiety or obsessive compulsive symptoms^20,38^ and some studies finding elevated depressive, anxiety (i.e. panic attacks) and post-traumatic stress disorder symptoms in particular,^14,15,22,39,40,41,42^ whereas other studies report no association between depressive or anxiety symptoms and SUDs.^27,43^ Further, suicidality has been found to be elevated in SMI/SUD patients in several studies,^20,27,30,43^ but not all.^13^

We aimed to determine the prevalence, and demographic and clinical correlates of co-occurring SUDs in a clinically heterogeneous sample of patients with psychotic disorders. In addition, we aimed to determine in SMI the association of co-occurring SUDs with anxiety, depressive symptoms including suicidality, involvement with police arrests, and prior treatment for SUDs.

## Methods

### Sample and Setting

We conducted a secondary analysis of a database (*N* = 248) derived from three separate studies that ran concurrently at Valkenberg hospital, a large psychiatric academic hospital attached to the University of Cape Town’s Department of Psychiatry and Mental Health. The hospital is a secondary and tertiary level service that receives referrals from 5 community-based regional psychiatric short-stay units and several psychiatry outpatient clinics situated in a number of community mental health centres within the Western and Southern Cape regions of South Africa. Participants attended inpatient or outpatient services or community psychiatric clinics in the hospitals’ Western metropolitan region catchment area. Participants from the first cross-sectional study; “Presentation and risk factor in the psychobiology of psychosis” (*N* = 86) were selected randomly from inpatients listed as attending pre-discharge inpatients wards.^44^ Inclusion criteria included a diagnosis of a psychotic disorder (schizophrenia, schizophreniform disorder, schizoaffective disorder, brief psychotic disorder, substance induced psychotic disorder, psychotic disorder not-otherwise specified) or bipolar I disorder with psychotic features. Diagnoses were based on DSM-IV-TR criteria (diagnostic codes 295.xx, 295.40, 295.70, 298.8, 291.xx, 292.xx, 298.9, 296.xx).^45^ All participants had to be conversational in the English language. Exclusion criteria included psychotic disorders due to a general medical condition, dementia, intellectual disability, a primary diagnosis of a personality disorder and neurological disorders.

The second study was a pilot randomised controlled trial (“Social inclusion Project-SIP”, *N* = 59) investigating the effect of a treatment partner text messaging and psychoeducational intervention on treatment adherence in patients with serious mental illness, and participants included inpatients attending the pre-discharge wards at Valkenberg hospital.^46^ The third study recruited outpatients via referral from clinicians and advertisements in the media and investigated the cortical inhibition and attentional modulation using MRI neuroimaging and electroencephalography across patients with schizophrenia, methamphetamine psychosis, and bipolar mood disorder with psychosis and normal controls (CIAM study, *N* = 103).^47^

Exclusion criteria for the last two studies were the same as the first study.

### Measures and procedures:

A socio-demographic schedule was used across the parent studies to record participants’ demographic details such as age, sex, self-identified ethnicity, level of education, marital status, employment and past drug or alcohol treatment. For self-identified ethnic groups the terms ‘mixed race (coloured)’ ‘black’ and ‘caucasian’ and ‘other’ (Asian), were not intended to reify sociocultural constructs but were instead used to study ongoing health disparities. Across all three studies all participants had to complete the English language version of the Structured Clinical Interview for DSM-IV (SCID-I),^48^ resulting in complete data across studies for this instrument. The SCID-I assesses mood episodes (depression and suicidality) in modules A, psychotic symptoms in module B and derives diagnoses for principal psychotic mood and psychotic disorders in modules C and D. Furthermore, lifetime SUDs were assessed using module E of the SCID-I which assesses abuse and dependence according to DSM-IV criteria. Anxiety including panic, specific and social anxiety, generalised anxiety, agoraphobia, obsessive compulsive and post-traumatic stress symptoms and disorders were assessed in module F of the SCID-I. The SCID-I assesses symptoms as either present or absent at threshold allows clinicians to rate symptoms as subthreshold based on clinical judgement in cases where a symptom appears to be present but falls short of the full criterion. From module A of the SCID-I, we coded the presence of any lifetime major depressive episode (MDE) as well as the presence of any lifetime threshold or subthreshold depressive symptoms defined as the presence of depressed mood or anhedonia, the minimum entry requirements for any (depressive) mood episode in the SCID-I. We entered suicidality as a separate variable in models. For anxiety, in addition to syndrome level disorders we entered the presence of any subthreshold or threshold symptoms that fell short of meeting criteria for a clinical disorder. We categorised depressive, anxiety, obsessive-compulsive and post-traumatic disorders or symptoms separately into different categories, with the presence of a disorder or symptoms coded = 1 and absence = 0. Panic, agoraphobia without a history of panic, social phobia, specific phobia and generalised anxiety were classified together as ‘lifetime anxiety disorders/ symptoms’. Obsessive-compulsive disorder and symptoms were classified separately and post-traumatic stress disorder and symptoms also into different categories. We assessed lifetime police contact and history of criminal arrests based on the section of the Addiction Severity Index (ASI) gathering information on legal involvement.^49^ Legal involvement and police arrests were classified as ‘serious violent crime’ (including assault, rape, murder or armed robbery), ‘major crime’ (shoplifting, vandalism, parole violation, forgery, weapons offense, burglary, arson, contempt of court, domestic violence not involving assault), ‘other crimes’ (possession of illegal substances, weapons offense, prostitution, disorderly conduct in public, major driving violation, driving under the influence of substances). SCID-I interviews typically lasted 2.5-3.5 hours. Additional information was considered in the diagnostic process from referral notes, including urine drug tests conducted on hospital admissions where available, past and current clinical records, interviews with other members of the multidisciplinary teams and information from family members or other associates of the patients. Participants were assessed by psychiatric nurses, research and senior psychiatrists with extensive training and experience in SCID-I interviewing. Inter-rater reliability was obtained on a smaller sample (*n* = 8) of participants with good agreement for the principle psychiatric diagnosis (kappa = 0.70, *p* < 0.001) and comorbid SUDs (kappa = 0.80, *p* < 0.001).

### Statistical analysis

We calculated the 95% confidence intervals of prevalence’s using the normal approximation of the binomial distribution. We constructed six separate dichotomous dependent variables denoting the presence or absence of any lifetime, alcohol, cannabis, methamphetamine, methaqualone and other drug (cocaine and hallucinogens) SUDs (abuse or dependence). We then explored the distribution and relationship between the different SUDs using logistic regression analyses. For the association between SUDs and demographic and clinical variables, we firstly conducted bivariate logistic regression analyses for each dependent variable separately onto each of the different independent demographic and clinical predictor variables. We then constructed multivariable logistic regression models with the dependent variables the various SUDs (any SUDS, alcohol, cannabis, methamphetamine, methaqualone, and other drug use disorders) and entered independent variables that were significant in the bivariate analyses at a *p* ≤ 0.10 levels into the final models.

We report the associations between independent variables and dependent variables as adjusted odds ratios (ORs) with their 95% confidence intervals. All analyses were two-tailed and considered significant at the 5% level. We used Stata version 13 for Windows for all analyses.^50^

### Ethical consideration

All participants in the original studies provided written informed consent to participate in the studies and the secondary data analysis was also approved by the Human Research Ethics Committee of the University of Cape Town (HREC 652/2014) with its contributory studies: PRP study (HREC: 332/2008) the SIP study (HREC: 511/2011) and the CIAM study (HREC: 192/2010).

## Results

### Sample characteristics

In the total of sample of 248 participants the mean age was 31.5 years (SD = 9.2), with the majority of participants being male (64.5%). Table 1 and 2 contain the sample demographic and clinical characteristics, respectively. Most participants were of ‘Coloured’ ethnic background, single, had less than 12 years of education, were unemployed and had a diagnosis of a schizophrenia spectrum disorder. Lifetime major depressive episodes or anxiety disorders occurred only in 20.6% and 13% of participants, but both depressive and anxiety symptoms were more prevalent and occurred in as many as half the sample (depressive symptoms, 55.7%) with anxiety symptoms occurring in a quarter (25%). Self-reported arrests were common and among participants with a SUD the most prevalent was arrest for ‘other crimes’ (26.1%) whereas arrests for major and serious violent crimes were less common (occurring in 10.1% and 11.6% with SUDs respectively). Of those patients with SUDs, less than a quarter (23.9%) indicated that they had attended any form of substance rehabilitation programme, of which 14.5% had attended inpatient and 13.0% outpatient rehabilitation programmes (Table 2).

**TABLE 1:** Sample sociodemographic characteristics (*N* = 248).

Sociodemographics	*N*	%
**Age (years)**		
18-29	122	49.2
30-44	97	39.1
45-65	29	11.7
**Sex**		
Female	88	35.5
Male	160	64.5
**Ethnicity**		
Mixed race (coloured)	141	56.9
Black	72	29.0
Caucasian	28	11.3
Other†	7	2.8
**Marital status**		
Never married	198	79.8
Married or cohabiting	33	13.3
Previously married	17	6.9
**Education (years)**		
≤ 7	39	15.7
8-11	119	48.0
12	64	25.8
> 12	26	10.5
**Employment**		
No	168	67.7
Yes	80	(32.3
**Study and setting**		
CIAM study (outpatients)	103	41.5
PRP study (inpatients)	86	34.7
SIP study (inpatients)	59	23.8

† , Other = Asian.

**TABLE 2:** Sample clinical characteristics (*N* = 248).

Clinical characteristics	*N*	%
**Diagnosis**		
Schizophrenia spectrum disorder†	132	53.2
Bipolar type I disorder	51	20.6
Schizoaffective disorder	33	13.3
Substance induced psychotic disorder	32	12.9
Lifetime MDE‡	51	20.6
Lifetime MDE‡ symptoms	138	55.7
**Suicidality**		
No suicidality	171	69.0
Ideation or plans	54	21.8
Attempt	23	9.3
Lifetime anxiety§ disorders	31	13
Lifetime anxiety§ symptoms	62	25
Lifetime OCD¶	2	0.8
Lifetime OCS¶	13	5.2
Lifetime PTSD††	10	4.0
Lifetime PTSD†† symptoms	23	9.3
**Legal involvement**‡‡		
No arrests	167	67.3
Serious/violent crime	22	8.9
Major crime	20	8.1
Other crime	39	15.7
**History of substance rehabilitation**		
Any rehabilitation	33	13.3
Inpatient	23	9.3
Outpatient	18	7.3

† , Schizophrenia, schizophreniform disorder, brief psychotic disorder, psychotic disorder NOS

‡ , MDE = Major depressive episode

§ , Symptoms or disorders: Panic disorder, Agoraphobia without a history of panic, specific phobia, social phobia, generalised anxiety disorder

¶ , OCD = Obsessive compulsive disorder. OCS = obsessive compulsive symptoms

†† , PTSD = Posttraumatic stress disorder.

‡‡ , Legal involvement: Serious violent crime (assault, rape, murder, armed robbery), Major crime (shoplifting, vandalism, parole violation, forgery, weapons offense, burglary, arson, contempt of court, domestic violence), Other crime (possession of illegal substances, weapons offense, prostitution, disorderly conduct in public, major driving violation, driving under the influence of substances).

### Prevalence, patterns and distribution of substance use disorders

In the total sample of 248 participants, the prevalence of any substance use disorder (abuse or dependence) was 55.6% (95% CI = 49.2% - 62.0%). For the individual SUDs in the total sample, the most common SUD was cannabis use disorders with a prevalence of 34.3% (95% CI: 28.3% - 40.5%), followed by alcohol use disorders (30.6%; 95% CI = 25.0% - 36.7%), methamphetamine use disorder (27.4%; 95% CI = 22.0% - 33.4%) and methaqualone (sedative-hypnotic) use disorders (10.4%; 95% CI = 6.9% - 14.9%). Other drug use disorders occurred at a much lower frequency with cocaine use disorder occurring in only 4.4% (95% CI = 2.2% - 7.7%) and hallucinogens (MDMA and LSD) only in 1.6% (95% CI = 0.4%- 4.0%). All participants with SUDs fulfilled criteria for more than one SUD, with 4% abusing more than one substance and 22.9% fulfilling criteria for more than one substance dependence syndrome. There was a significant association between having a cannabis and alcohol use disorder (OR = 2.0, *p* = 0.031, 95% CI = 1.1 – 3.7) (Table 3). In turn, cannabis, methamphetamine or methaqualone use disorders often occurred together with the odds of having any one of these disorders significantly increasing the odds of having another with as many as 4-5 fold (Table 3).

**TABLE 3:** Patterns and relationship between substance use disorders (*N* = 248).

SUD†	Alcohol (*R*^2^ = 0.05)		Cannabis (*R*^2^ = 0.17)		Methamphetamine (*R*^2^ = 0.17)		Methaqualone (*R*^2^ = 0.27)		Other (*R*^2^ = 0.13)	
	Adjusted OR	95% CI	Adjusted OR	95% CI	Adjusted OR	95% CI	Adjusted OR	95% CI	Adjusted OR	95% CI
Alcohol	-	-	2.0*	(1.0 - 3.7)	1.2	(0.6 - 2.5)	2.4	(1.0 - 6.2)	2.1	(0.6 - 7.5)
Cannabis	2.0*	(1.1 - 3.7)	-	-	4.4***	(2.3 - 8.5)	5.1**	(1.7 - 15.0)	1.9	(0.5 - 7.6)
Methamphetamine	1.3	(0.6 - 2.5)	4.4***	(2.3 - 8.4)	-	-	5.2**	(1.9 - 14.1)	2.0	(0.5 - 7.5)
Methaqualone	2.1	(0.8 - 5.2)	4.7**	(1.6 - 14.1)	4.7**	(1.7 - 12.8)	-	-	3.3	(0.8 - 13.7)
Other	2.1	(0.6 - 7.3)	1.8	(0.4 - 7.7)	1.7	(0.5 - 6.5)	3.7	(0.9 - 14.7)	-	-

*** *p* < 0.001,

** *p* < 0.01,

* *p* < 0.05.

† , SUD = substance use disorders.

Each substance in columns adjusted for the effects of all other substances.

*R*^2^ = general coefficient of determination, (McFadden’s *R*^2^).

### Demographic and clinical correlates of substance use disorders

After adjustment for demographic and clinical covariates in multivariable models some variables remained significantly associated with the presence of the various SUDs (Tables 4 and 5). For the presence of any SUD there were significant positive associations in the adjusted multivariable model with male sex, substance induced psychosis, serious violent and ‘other crimes’ categories. For alcohol use disorders there were significant positive associations with male sex, lifetime anxiety symptoms, suicide attempts and other crime categories. For cannabis use disorder we found significant positive associations with male sex, lower educational attainment, substance induced psychosis, major and other crimes. For methamphetamine use disorders we found significant associations with younger age, significant negative associations with black ethnicity as compared to mixed (“coloured”) ethnicity, a strong positive association with having a substance induce psychosis and a significant positive association with other crimes. For methaqualone there was a significant association between past marriage (divorced or widowed), and serious and violent crime, whereas for the category of ‘Other SUDs’ (of which cocaine 92%, LSD or MDMA 8%) there were significant associations between Asian ethnicity and serious violent crime.

**TABLE 4:** Adjusted demographic and clinical association with any, alcohol and cannabis use disorders. (***N*** = 248).

Variable	Any SUD (*R*^2^ = 0.26)		Alcohol (*R*^2^ = 0.16)		Cannabis (*R*^2^ = 0.24)	
	Adjusted OR	95% CI	Adjusted OR	95% CI	Adjusted OR	95% CI
**Age (years)**						
18-29(ref)	1	(ref)	1	(ref)	1	(ref)
30-44	0.9	(0.4 - 1.8)	0.8	(0.4 - 1.5)	0.6	(0.3 - 1.1)
45-65	0.6	(0.2 - 1.9)	1.0	(0.3 - 3.5)	1.0	(0.3 - 3.1)
**Sex**						
Male:Female	3.9***	(1.9 - 8.3)	2.8**	(1.3 - 6.0)	4.7***	(2.0 - 11.1)
**Ethicity**						
Mixed race (coloured) (ref)	-	-	-	-	1	(ref)
Black	-	-	-	-	0.5	(0.2 - 1.1)
Caucasian	-	-	-	-	0.7	(0.2 - 2.2)
Other	-	-	-	-	1.2	(0.2 - 6.8)
**Education**						
≤ 7	1	(ref)	-	-	1	(ref)
8-11	0.6	(0.2 - 1.5)	-	-	0.5	(0.2 - 1.3)
12	0.6	(0.2 - 1.8)	-	-	0.4	(0.1 - 1.0)
> 12	0.6	(0.2 - 2.3)	-	-	0.1*	(0.0 - 0.8)
Employed	-	-	-	-	0.8	(0.4 - 1.7)
**Marital status**						
Never married	1	(ref)	1	(ref)	-	-
Married or cohabiting	0.7	(0.3 - 1.9)	0.7	(0.2 - 2.0)	-	-
Previously married	1.0	(0.3 - 3.8)	0.3	(0.1 - 1.7)	-	-
**Diagnosis**						
Schizophrenia spectrum disorder†	1	(ref)	-	-	1	(ref)
Bipolar type I disorder	1.1	(0.5 - 2.5)	-	-	1.7	(0.7 - 4.0)
Schizoaffective disorder	0.9	(0.3 - 2.4)	-	-	0.9	(0.3 - 2.9)
Substance induced psychotic disorder	12.0***	(3.3 - 43.6)	-	-	3.3*	(1.1 - 9.4)
Lifetime MDE	0.5	(0.2 - 1.2)	-	-	0.9	(0.4 - 2.4)
Lifetime MDE symptoms	-	-	1.6	(0.7 - 3.3)	-	-
Lifetime anxiety symptoms	-	-	2.5*	(1.2 - 5.1)	0.5	(0.2 - 1.1)
**Suicidality**						
No ideation	-	-	1	(ref)	-	-
Ideation or plan	-	-	1.0	(0.4 - 2.5)	-	-
Attempt	-	-	3.3*	(1.1 - 9.8)	-	-
**Legal involvement**						
No arrests	1	(ref)	1	(ref)	1	(ref)
Serious violent crime	3.2*	(1.0 - 9.8)	1.6	(0.6 - 4.5)	2.0	(0.7 - 5.7)
Major crime	2.7	(0.9 - 8.4)	2.3	(0.8 - 7.0)	3.5*	(1.2 - 10.2)
Other crime	11.9***	(3.4 - 42.2)	2.8*	(1.2 - 6.5)	3.4**	(1.5 - 8.0)
**Study and setting**						
CIAM study (outpatients)	-	-	1	(ref)	-	-
PRP study (inpatients)	-	-	1.6	(0.8 - 3.2)	-	-
SIP study (inpatients)	-	-	0.3*	(0.1 - 0.9)	-	-

*** *p* < 0.001,

** *p* < 0.01,

* *p* < 0.05.

† , Schizophrenia, schizophreniform disorder, brief psychotic disorder, psychotic disorder NOS.

*R*^2^, general coefficient of determination, (McFadden’s *R*^2^).

Omitted variables did not reach significance at *p* < 0.10, and were not entered into models.

**TABLE 5:** Adjusted demographic and clinical association with methamphetamine, metaqualone and other substance use disorders. (*N* = 248).

Variable	Methamphetamine (*R*^2^ = 0.38)		Methaqualone (*R*^2^ = 0.17)		Other (*R*^2^ = 0.29)	
	Adjusted OR	95% CI	Adjusted OR	95% CI	Adjusted OR	95% CI
**Age (years)**						
18-29(ref)	1	(ref)	-	-	-	-
30-44	0.7	(0.3 - 1.6)	-	-	-	-
45-65	0.1*	(0.0 - 0.7)	-	-	-	-
**Sex**						
Male:Female	1.9	(0.7 - 5.3)	2.6	(0.8 - 8.4)	2.7	(0.4 - 16.0)
**Ethnicity**						
Mixed race (coloured)	1	(ref)	-	-	1	(ref)
Black	0.3**	(0.1 - 0.7)	-	-	0.5	(0.1 - 3.3)
Caucasian	0.6	(0.2 - 2.4)	-	-	2.0	(0.4 - 9.5)
Asian	0.5	(0.1 - 4.5)	-	-	20.9**	(2.7 - 162.6)
**Education**						
≤ 7	1	(ref)	-	-	-	-
8-11	0.7	(0.2 - 2.0)	-	-	-	-
12	0.5	(0.1 - 1.5)	-	-	-	-
> 12	0.2	(0.0 - 1.8)	-	-	-	-
Employed	0.7	(0.3 - 1.7)	-	-	-	-
**Marital status**						
Never married	-	-	1	(ref)	-	-
Married or cohabiting	-	-	0.4	(0.1 - 2.5)	-	-
Previously married	-	-	4.4*	(1.1 - 16.6)	-	-
**Diagnosis**						
Schizophrenia spectrum disorder†	1	(ref)	-	-	-	-
Bipolar type I disorder	0.5	(0.2 - 1.6)	-	-	-	-
Schizoaffective disorder	0.4	(0.1 - 1.6)	-	-	-	-
Substance induced psychotic disorder	26.5***	(7.1 - 98.6)	-	-	-	-
Lifetime MDE	0.6	(0.3 - 1.2)	0.4	(0.2 - 1.0)	-	-
Lifetime anxiety symptoms	-	-	-	-	2.7	(0.8 - 9.3)
**Legal involvement**						
No arrests	1	(ref)	1	(ref)	1	(ref)
Serious violent crime	2.9	(0.9 - 9.7)	4.4*	(1.3 - 15.1)	6.7*	(1.4 - 32.0)
Major crime	1.0	(0.2 - 4.1)	2.4	(0.7 - 8.8)	0.2	(0.0 - 10.1)
Other crime	4.8**	(1.8 - 12.8)	1.3	(0.4 - 4.1)	3.4	(0.8 - 14.5)
**Study and setting**						
CIAM study (outpatients)	-	-	1	(ref)	-	-
PRP study (inpatients)	-	-	3.3*	(1.1 - 10.0)	-	-
SIP study (inpatients)	-	-	2.5	(0.7 - 8.6)	-	-

*** *p* < 0.001,

** *p* < 0.01,

* *p* < 0.05.

† , Schizophrenia, schizophreniform disorder, brief psychotic disorder, psychotic disorder NOS.

*R*^2^ = general coefficient of determination, (McFadden’s *R*^2^).

Omitted variables did not reach significance at *p* < 0.10, and were not entered into models.

## Discussion

### Main findings and comparison with other studies

In this study, one of the few from a LMIC context such as South Africa, we confirmed the high prevalence of SUDs in patients with a range of different psychotic disorders. We found prevalence for any SUD of 55.6%, similar to other studies in similar populations across the world, and very close to the previously found prevalence of 51% in a similar sample from Cape Town.^3^ Similar to other studies, including meta-analyses, we found the predominant substances in this sample were cannabis then alcohol, followed by methamphetamines.^1,3,4,5,6^ In contrast to other studies we found a lower prevalence of cocaine use disorders and no participants had opioid use disorders.^34,36^ One reason for the lower prevalence of cocaine use (and higher methamphetamine use) in this sample may be the lower socioeconomic status of in our sample with most patients being unemployed. Another reason for the low occurrence of SUDs such as cocaine and heroin may be the fact as our sample consisted exclusively of patients with psychotic disorders, the majority with a diagnosis of schizophrenia spectrum disorders, which is consistent with other samples for other settings that also included mainly this patient group and excluded patients with a diagnosis of mood disorders without psychotic features and patients with principle diagnosis of personality disorders (the latter who may be more likely to use opioids)^34^ Our finding of 23.7% of participants reporting having attended substance rehabilitation programmes is higher than the previously found in another study from a Cape Town with serious mental illness, where less than 5% reported such interventions.^3^ Nevertheless, this is still less than a quarter of the total sample.

Consistent with other studies the association between methamphetamine and younger age remained significant in the multivariable models.^18^ As in most studies in adults,^1,3,15, 21,26^,^27,28,29,30,31,33,36,40^ with the exception of one study in older adolescents,^20^ we found a significant association between male sex and SUDs across most substances in the adjusted analyses. We found a significant association between having been previously married (i.e. separated or divorced, widowed) and methaqualone use. Similar to other studies, none of the other SUDs in our sample were associated with marital status,^19^ black participants were significantly less likely to use methamphetamine compared to participants from a mixed (coloured) ethnic background. Some studies from the United States of America have found a significant positive relationship between ethnic minorities (i.e. African Americans), and substance use; amphetamine and cocaine use in particular.^10,25,31^ Comparatively high use of methamphetamines in mixed ethnic groups versus other ethnic groups has also been found in other studies in non-psychotic populations in the South African context,^51^ and may reflect a neighbourhood effect as population groups still correlate with geographical areas a result of the legacy of Apartheid segregation in South Africa. Similar to other studies,^26,28,31,36^ we found a significant association between lower educational attainment and any SUDs in unadjusted analyses, which remained significant for only cannabis use disorders in the adjusted analyses. One reason for this in populations with predominantly schizophrenia patients has been postulated to be school drop-out associated with cannabis use.^28^

Similarly to other studies we found a significant association between SUD and a diagnosis of substance induced psychosis.^3,52^ Depressive and anxiety symptoms were significantly more prevalent in participants with alcohol use disorders, and anxiety symptoms remained significant in the adjusted analysis for alcohol use disorders. These findings are consistent with other studies that have found higher anxiety symptoms (i.e. panic) in patients with alcohol use disorders but not in those with cannabis use disorders.^14^ There is however some inconsistency in the literature; several studies have found depressive and anxiety symptoms to occur more often in both cannabis and alcohol or any SUDs categories,^15,16,22,39^ but some studies have shown no association with depressive or anxiety symptoms^19^ or even lower depressive and anxiety symptoms especially in predominately cannabis using populations.^20^ Similar to other studies we found a significant adjusted association with suicide attempts,^30,43^ particularly for participants with alcohol use disorders.

With the exception of alcohol use disorder, all categories of SUDs including any SUDs had significantly elevated occurrence of legal involvement, including serious and violent crime with an even stronger association with the ‘other crime’ category denoting police arrests for crimes relating to illegal drug possession, prostitution, driving violations and disorderly conduct in public. In addition, cannabis users also were significantly more likely to get arrested for major crimes (shoplifting, vandalism, parole violation, forgery, weapons offense, burglary, arson, domestic violence not involving assault) and methaqualone and ‘other drug users’ (predominantly cocaine users) were significantly more likely to be involved with serious violent offenses (assault, rape, murder, armed robbery). These findings are consistent with those from high income countries^1,13,30,34,36^ and among the first from a LMIC context. Importantly, although risky and impulsive behaviour manifested as suicide attempts in alcohol users, other drug users were more likely to engage in externalising risk-taking behaviours involving criminal activity in the adjusted analyses.

### Implications for clinicians

Our results confirm the clinical profile of participants with SUDs as being more likely to be male, have a younger age (in particular methamphetamine users) and having a diagnosis of a substance induced psychosis. In particular, those with alcohol use disorders were more likely to experience anxiety symptoms (i.e. panic, generalised and social anxiety) and significantly more likely to have attempted suicide. This underscores the importance of screening for anxiety and suicide risk assessments in patients with co-occurring alcohol use disorders. For most substances, involvement in crimes relating to drug possession, prostitution, disorderly conduct and driving violations (‘other crimes’ category) were significantly more likely; and involvement in major crimes, serious violent crimes was also significantly elevated for cannabis, methaqualone and other (predominantly cocaine) users. Practitioners who manage patients with co-occurring disorder are likely to need to liaise with criminal justice institutions, state prosecutors and police.

### Study strengths and limitations

Several limitations should be acknowledged. First, is the absence of biological verification of substance use.

Although urine tests for substances such as cannabis and methamphetamine conducted on some patients who were admitted to short-stay psychiatric units were taken into consideration during patient assessment and interviews, tests for alcohol and other drugs are not routinely conducted, neither were such tests recorded consistently so as to allow for use in this study. Short detection windows of most biological tests are also likely to result in false negative tests in participants who used recently but were tested only days after last use. As participants are more likely to underreport substances this would have led to an underestimation of substance use in this study. In addition, self-report of substances have been shown to yield accurate results, with other techniques like hair samples often being problematic in multi-ethnic samples.^53^ Self-report is also characteristic of epidemiological studies in this area.^18^ Second is the reliance on self-report, family collateral and past records to record criminal involvement. We lacked police data on formal charges and conviction rates and our data was limited to reasons for police arrests. As the design of the some of the parent studies were cross-sectional, recall bias could have affected reports of substance use and police arrest rates. Third, the use of DSM hierarchical rules may lead to lower prevalence estimates for anxiety, obsessive-compulsive and post-traumatic stress disorders. Nevertheless, we also recorded subthreshold anxiety, obsessive and post-traumatic stress symptoms, which led to higher yields of these symptoms. It is also not possible to determine the direction of the association between alcohol use and anxiety symptoms as alcohol withdrawal can be associated with anxiety but is also possible that people with more anxiety are more likely to use alcohol. Finally, we did not have continuous measures in this sample of severity rating to psychotic symptoms or anxiety and depressive symptoms (i.e. PANSS scales).

## Conclusion

This study found high prevalence of substance use disorders and multiple substance use in patients with psychotic disorders in a LMIC context. This underscores the importance of a thorough clinical assessment for various substance use disorders, and when present, for anxiety, suicidality and risky behaviours involving clashes with the law.

## References

[1] Cantor-Graae E, Nordstrom LG, McNeil TF Substance abuse in schizophrenia: a review of the literature and a study of correlates in Sweden. Schizophrenia research. 2001;48(1):69–82. 10.1016/S0920-9964(00)00114-6 11278155

[2] Lambert M, Conus P, Lubman DI, Wade D, Yuen H, Moritz S, et al The impact of substance use disorders on clinical outcome in 643 patients with first-episode psychosis. Acta psychiatrica Scandinavica. 2005;112(2):141–8. 10.1111/j.1600-0447.2005.00554.x 15992396

[3] Weich L, Pienaar W Occurrence of comorbid substance use disorders among acute psychiatric inpatients at Stikland Hospital in the Western Cape, South Africa. African journal of psychiatry. 2009;12(3):213–7. 10.4314/ajpsy.v12i3.48496 19750250

[4] Koskinen J, Lohonen J, Koponen H, Isohanni M, Miettunen J Prevalence of alcohol use disorders in schizophrenia–a systematic review and meta-analysis. Acta psychiatrica Scandinavica. 2009;120(2):85–96. 10.1111/j.1600-0447.2009.01385.x 19374633

[5] Koskinen J, Lohonen J, Koponen H, Isohanni M, Miettunen J Rate of cannabis use disorders in clinical samples of patients with schizophrenia: a meta-analysis. Schizophrenia bulletin. 2010;36(6):1115–30. 10.1093/schbul/sbp031 19386576 PMC2963055

[6] Sara GE, Large MM, Matheson SL, Burgess PM, Malhi GS, Whiteford HA, et al Stimulant use disorders in people with psychosis: a meta-analysis of rate and factors affecting variation. The Australian and New Zealand journal of psychiatry. 2015;49(2):106–17. 10.1177/0004867414561526 25518844

[7] Swofford CD, Kasckow JW, Scheller-Gilkey G, Inderbitzin LB Substance use: a powerful predictor of relapse in schizophrenia. SchizophrRes. 1996;20(1–2):145–151. 10.1016/0920-9964(95)00068-2 8794502

[8] Wade D, Harrigan S, McGorry PD, Burgess PM, Whelan G Impact of severity of substance use disorder on symptomatic and functional outcome in young individuals with first-episode psychosis. JClinPsychiatry. 2007;68(5):767–74. 10.4088/JCP.v68n0517 17503988

[9] Mazzoncini R, Donoghue K, Hart J, Morgan C, Doody GA, Dazzan P, et al Illicit substance use and its correlates in first episode psychosis. Acta psychiatrica Scandinavica. 2010;121(5):351–8. 10.1111/j.1600-0447.2009.01483.x 19824986

[10] Montross LP, Barrio C, Yamada AM, Lindamer L, Golshan S, Garcia P, et al Tri-ethnic variations of co-morbid substance and alcohol use disorders in schizophrenia. Schizophrenia research. 2005;79(2–3):297–305. 10.1016/j.schres.2005.04.014 15978782

[11] Weaver T, Rutter D, Madden P, Ward J, Stimson G, Renton A Results of a screening survey for co-morbid substance misuse amongst patients in treatment for psychotic disorders: prevalence and service needs in an inner London borough. Social psychiatry and psychiatric epidemiology. 2001;36(8):399–406. 10.1007/s001270170030 11766970

[12] Dixon L, Haas G, Weiden PJ, Sweeney J, Frances AJ Drug abuse in schizophrenic patients: clinical correlates and reasons for use. The American journal of psychiatry. 1991;148(2):224–30. 1987823 10.1176/ajp.148.2.224

[13] Fowler IL, Carr VJ, Carter NT, Lewin TJ Patterns of current and lifetime substance use in schizophrenia. Schizophrenia bulletin. 1998;24(3):443–55. 10.1093/oxfordjournals.schbul.a033339 9718636

[14] Goodwin RD, Amador XF, Malaspina D, Yale SA, Goetz RR, Gorman JM Anxiety and substance use comorbidity among inpatients with schizophrenia. Schizophrenia research. 2003;61(1):89–95. 10.1016/S0920-9964(02)00292-X 12648739

[15] Jimenez-Castro L, Hare E, Medina R, Raventos H, Nicolini H, Mendoza R, et al Substance use disorder comorbidity with schizophrenia in families of Mexican and Central American ancestry. Schizophrenia research. 2010;120(1–3):87–94. 10.1016/j.schres.2010.02.1053 20303714 PMC2940120

[16] Margolese HC, Carlos Negrete J, Tempier R, Gill K A 12-month prospective follow-up study of patients with schizophrenia-spectrum disorders and substance abuse: changes in psychiatric symptoms and substance use. Schizophrenia research. 2006;83(1):65–75. 10.1016/j.schres.2005.11.019 16460917

[17] Mueser KT, Yarnold PR, Levinson DF, Singh H, Bellack AS, Kee K, et al Prevalence of substance abuse in schizophrenia: demographic and clinical correlates. Schizophrenia bulletin. 1990;16(1):31–56. 10.1093/schbul/16.1.31 2333480

[18] Sara GE, Burgess PM, Malhi GS, Whiteford HA, Hall WC Stimulant and other substance use disorders in schizophrenia: prevalence, correlates and impacts in a population sample. The Australian and New Zealand journal of psychiatry. 2014;48(11):1036–47. 10.1177/0004867414533838 24819935

[19] Sevy S, Robinson DG, Holloway S, Alvir JM, Woerner MG, Bilder R, et al Correlates of substance misuse in patients with first-episode schizophrenia and schizoaffective disorder. Acta psychiatrica Scandinavica. 2001;104(5):367–74. 10.1111/j.1600-0447.2001.00452.x 11722318

[20] Shoval G, Zalsman G, Apter A, Diller R, Sher L, Weizman A A 10-year retrospective study of inpatient adolescents with schizophrenia/schizoaffective disorder and substance use. Comprehensive psychiatry. 2007;48(1):1–7. 10.1016/j.comppsych.2006.05.002 17145274

[21] Escamilla MA, Batki S, Reus VI, Spesny M, Molina J, Service S, et al Comorbidity of bipolar disorder and substance abuse in Costa Rica: pedigree- and population-based studies. Journal of affective disorders. 2002;71(1–3):71–83. 10.1016/S0165-0327(01)00373-1 12167503

[22] Kerfoot KE, Rosenheck RA, Petrakis IL, Swartz MS, Keefe RS, McEvoy JP, et al Substance use and schizophrenia: adverse correlates in the CATIE study sample. Schizophrenia research. 2011;132(2–3):177–82. 10.1016/j.schres.2011.07.032 21872443

[23] Mowbray CT, Ribisl KM, Solomon M, Luke DA, Kewson TP Characteristics of dual diagnosis patients admitted to an urban, public psychiatric hospital: an examination of individual, social, and community domains. The American journal of drug and alcohol abuse. 1997;23(2):309–26. 10.3109/00952999709040949 9143641

[24] Bersani G, Orlandi V, Kotzalidis GD, Pancheri P Cannabis and schizophrenia: impact on onset, course, psychopathology and outcomes. European archives of psychiatry and clinical neuroscience. 2002;252(2):86–92. 10.1007/s00406-002-0366-5 12111342

[25] Compton WM 3rd, Cottler LB, Ben Abdallah A, Phelps DL, Spitznagel EL, Horton JC Substance dependence and other psychiatric disorders among drug dependent subjects: race and gender correlates. The American journal on addictions. 2000;9(2):113–25. 10.1080/10550490050173181 10934573

[26] Hapangama A, Kuruppuarachchi KA, Pathmeswaran A Substance use disorders among mentally ill patients in a General Hospital in Sri Lanka: prevalence and correlates. The Ceylon medical journal. 2013;58(3):111–5. 10.4038/cmj.v58i3.6103 24081171

[27] Kamali M, Kelly L, Gervin M, Browne S, Larkin C, O’Callaghan E The prevalence of comorbid substance misuse and its influence on suicidal ideation among in-patients with schizophrenia. Acta psychiatrica Scandinavica. 2000;101(6):452–6. 10.1034/j.1600-0447.2000.101006452.x 10868468

[28] Kavanagh DJ, Waghorn G, Jenner L, Chant DC, Carr V, Evans M, et al Demographic and clinical correlates of comorbid substance use disorders in psychosis: multivariate analyses from an epidemiological sample. Schizophrenia research. 2004;66(2–3):115–24. 10.1016/S0920-9964(03)00130-0 15061243

[29] Latt N, Jurd S, Tennant C, Lewis J, Macken L, Joseph A, et al Alcohol and substance use by patients with psychosis presenting to an emergency department: changing patterns. Australasian psychiatry : bulletin of Royal Australian and New Zealand College of Psychiatrists. 2011;19(4):354–9. 10.3109/10398562.2011.579971 21851228

[30] Rush B, Koegl CJ Prevalence and profile of people with co-occurring mental and substance use disorders within a comprehensive mental health system. Canadian journal of psychiatry Revue canadienne de psychiatrie. 2008;53(12):810–21. 10.1177/070674370805301207 19087479

[31] Swartz MS, Wagner HR, Swanson JW, Stroup TS, McEvoy JP, Canive JM, et al Substance use in persons with schizophrenia: baseline prevalence and correlates from the NIMH CATIE study. The Journal of nervous and mental disease. 2006;194(3):164–72. 10.1097/01.nmd.0000202575.79453.6e 16534433

[32] Talamo A, Centorrino F, Tondo L, Dimitri A, Hennen J, Baldessarini RJ Comorbid substance-use in schizophrenia: relation to positive and negative symptoms. Schizophrenia research. 2006;86(1–3):251–5. 10.1016/j.schres.2006.04.004 16750347

[33] Van Mastrigt S, Addington J, Addington D Substance misuse at presentation to an early psychosis program. Social psychiatry and psychiatric epidemiology. 2004;39(1):69–72. 10.1007/s00127-004-0713-0 15022049

[34] Carra G, Crocamo C, Borrelli P, Popa I, Ornaghi A, Montomoli C, et al Correlates of dependence and treatment for substance use among people with comorbid severe mental and substance use disorders: findings from the “Psychiatric and Addictive Dual Disorder in Italy (PADDI)” Study. Comprehensive psychiatry. 2015;58:152–9. 10.1016/j.comppsych.2014.11.021 25591906

[35] McCabe PJ, Christopher PP, Druhn N, Roy-Bujnowski KM, Grudzinskas AJ Jr., Fisher WH Arrest types and co-occurring disorders in persons with schizophrenia or related psychoses. The journal of behavioral health services & research. 2012;39(3):271–84. 10.1007/s11414-011-9269-4 22270830

[36] Mueser KT, Yarnold PR, Rosenberg SD, Swett C Jr., Miles KM, Hill D Substance use disorder in hospitalized severely mentally ill psychiatric patients: prevalence, correlates, and subgroups. Schizophrenia bulletin. 2000;26(1):179–92. 10.1093/oxfordjournals.schbul.a033438 10755680

[37] Robertson AG, Swanson JW, Frisman LK, Lin H, Swartz MS Patterns of justice involvement among adults with schizophrenia and bipolar disorder: key risk factors. Psychiatric services (Washington, DC). 2014;65(7):931–8. 10.1176/appi.ps.201300044 24633645

[38] Seedat F, Roos JL, Pretorius HW, Karayiorgou M, Nel B Prevalence and clinical characteristics of obsessive-compulsive disorder and obsessive compulsive symptoms in Afrikaner schizophrenia and schizoaffective disorder patients. AfrJPsychiatry (Johannesbg). 2007;10(4):219–24. 10.4314/ajpsy.v10i4.30259 19588030

[39] Cuffel BJ, Heithoff KA, Lawson W Correlates of patterns of substance abuse among patients with schizophrenia. Hospital & community psychiatry. 1993;44(3):247–51. 10.1176/ps.44.3.247 8444435

[40] Margolese HC, Malchy L, Negrete JC, Tempier R, Gill K Drug and alcohol use among patients with schizophrenia and related psychoses: levels and consequences. Schizophrenia research. 2004;67(2–3):157–66. 10.1016/S0920-9964(02)00523-6 14984874

[41] O’Hare T, Sherrer M Lifetime trauma, subjective distress, substance use, and PTSD symptoms in people with severe mental illness: comparisons among four diagnostic groups. Community mental health journal. 2013;49(6):728–32. 10.1007/s10597-013-9620-8 23812793

[42] Scheller-Gilkey G, Moynes K, Cooper I, Kant C, Miller AH Early life stress and PTSD symptoms in patients with comorbid schizophrenia and substance abuse. Schizophrenia research. 2004;69(2–3):167–74. 10.1016/S0920-9964(03)00188-9 15469190

[43] Gut-Fayand A, Dervaux A, Olie JP, Loo H, Poirier MF, Krebs MO Substance abuse and suicidality in schizophrenia: a common risk factor linked to impulsivity. Psychiatry research. 2001;102(1):65–72. 10.1016/S0165-1781(01)00250-5 11368841

[44] Shelly J, Uhlmann A, Sinclair H, Howells FM, Sibeko G, Wilson D, et al First-Rank Symptoms in Methamphetamine Psychosis and Schizophrenia. Psychopathology. 2016;49(6):429–35. 10.1159/000452476 27926911

[45] American Psychiatric Association Diagnostic and Statistical Manual of Mental Disorders. 4th ed. Washington, DC: American Psychiatric Association; 2000.

[46] Mall S, Sibeko G, Temmingh H, Stein DJ, Milligan P, Lund C Using a treatment partner and text messaging to improve adherence to psychotropic medication: a qualitative formative study of service users and caregivers in Cape Town, South Africa. African journal of psychiatry. 2013;16(5):364–70. 10.4314/ajpsy.v16i5.49 24051670

[47] Howells FM, Temmingh HS, Hsieh JH, van Dijen AV, Baldwin DS, Stein DJ Electroencephalographic delta/alpha frequency activity differentiates psychotic disorders: a study of schizophrenia, bipolar disorder and methamphetamine-induced psychotic disorder. Translational psychiatry. 2018;8(1):75 10.1038/s41398-018-0105-y 29643331 PMC5895848

[48] First M, RL S, M G, JBW W Structured Clinical Interview for DSM-IV Axis I disorders-Patient Edition (SCID-I-P, version 2). 1994 1994.

[49] McLellan AT, Kushner H, Metzger D, Peters R, Smith I, Grissom G, et al The Fifth Edition of the Addiction Severity Index. JSubstAbuse Treat. 1992;9(3):199–213. 10.1016/0740-5472(92)90062-S 1334156

[50] StataCorp *Stata Statistical Software: Release 13*. College Station, TX: StataCorp LP 2013.

[51] Myers B, Kline TL, Browne FA, Carney T, Parry C, Johnson K, et al Ethnic differences in alcohol and drug use and related sexual risks for HIV among vulnerable women in Cape Town, South Africa: implications for interventions. BMC public health. 2013;13:174 10.1186/1471-2458-13-174 23442318 PMC3598514

[52] Helseth V, Lykke-Enger T, Johnsen J, Waal H Substance use disorders among psychotic patients admitted to inpatient psychiatric care. Nordic journal of psychiatry. 2009;63(1):72–7. 10.1080/08039480802450439 19034727

[53] Selten JP, Bosman IJ, de Boer D, Veen ND, van der Graaf Y, Maes RA, et al Hair analysis for cannabinoids and amphetamines in a psychosis incidence study. European neuropsychopharmacology : the journal of the European College of Neuropsychopharmacology. 2002;12(1):27–30. 10.1016/S0924-977X(01)00129-8 11788237

